# Satisfaction and perceived impact of virtual learning during COVID‐19 lockdown: A case study of an online nursing research conference

**DOI:** 10.1002/nop2.1857

**Published:** 2023-05-31

**Authors:** Emmanuel O. Adesuyi, Oluwadamilare Akingbade, Yetunde O. Tola, Oluwadamilola Otun, Damilola M. Faleti, Israel O. Fawole, Daniel D. Faleti, Emmanuel A. Dairo, Olamide Sado, Pelumi Adefehinti, Taiwo Olubunmi Adewa

**Affiliations:** ^1^ Adult Nursing Department, School of Nursing and Midwifery Birmingham City University Birmingham UK; ^2^ The Nethersole School of Nursing, Faculty of Medicine Chinese University of Hong Kong Hong Kong China; ^3^ Institute of Nursing Research Osogbo Nigeria; ^4^ Babcock University Teaching Hospital Ilishan‐Remo Nigeria

**Keywords:** COVID‐19, learning, nursing education, online learning, teaching, virtual learning

## Abstract

**Aim:**

This study aimed to assess nurses' satisfaction and perceptions of the impact of virtual learning.

**Design:**

A descriptive cross‐sectional survey.

**Method:**

236 nurses attending an online conference from several parts of Nigeria participated in the study. Analysed data were summarized and presented in tables and graphs, while linear regression was used to measure the associations.

**Results:**

Most of the respondents perceived the programme as highly impactful. All three domains: learner‐content interaction (*p* = 0.020), learner–instructor interaction (*p* = 0.000) and learner–learner interaction (*p* = 0.000), were found to be statistically significantly associated with the perceived impact of the programme, and thus statistically significant predictors of the effects of online learning (*p* = 0.02), (*F* = 5.471). Conclusively, the Interaction of learners with learning content, lecturers and other learners was seen as determinants of an effective and impactful online education. It is recommended that nursing training institutions embrace online learning either as the leading platform or as an adjunct to a face‐to‐face method.

## 1 INTRODUCTION

Recent global happenings seem to have caused a paradigm shift in knowledge delivery in the educational sector from the conventional system to virtual learning, otherwise known as e‐learning. More so, the ravaging impact of the coronavirus, also called COVID‐19, has placed more demands on technology for an alternative means of learning virtually (Adeoye et al., 2020). United Nations Educational, Scientific and Cultural Organization UNESCO (2020) claimed that more than 91% of the world's student population were affected because almost all educational institutions were closed due to the COVID‐19 pandemic. Students and educators at all levels of the academic institution have felt the impact of this pandemic on its emergence as a public health emergency. Institutions and organizations in Africa began to explore virtual learning platforms in a bid to cope and adapt to the resultant change from the shock initiated by the disruption of academic activities as some of the institutions were at the beginning of the semester, preparing for examinations, admitting first‐year students etc. (Egoigwe, 2020).

Virtual learning refers to an educationally inspired technology that uses electronic or computerized platforms to acquire knowledge at the convenience of both the learners and lecturers. It is invariably associated with the holistic incorporation of modern telecommunication equipment and Information and Communication Technology (ICT) resources into the education system (Eze et al., 2018). Several authors have explored virtual learning by considering various paradigms, including distance learning, e‐learning and online learning (Avelino et al., 2017; Bolliger & Wasilik, 2009; Deepa et al., 2022; Murray & Curran, 2008). Park (2009) claimed that the word ‘e’ in ‘e‐learning’ should refer to ‘everything, everyone, engaging and easy’ in addition to ‘electronic’. Increased level of confidence for learners and lecturers, better content delivery and interactivity are benefits of virtual learning (Adeoye et al., 2020).

Before the COVID‐19 pandemic, there has been ongoing regard for the use of technology to advance higher education (Vela, 2018). Virtual learning was regularly considered as an essential instrument for educational improvement, which in most cases requires onerous teaching and learning techniques to complement the conventional method in Nigerian institutions (Kochar & Samad, 2018; Serbessa, 2006). Whereas e‐learning is widely used in numerous higher institutions of the world (Eze et al., 2018). Eduventure (2019) reported that 15% of all undergraduate students in the US enrolled for online and distance learning. However, in Nigeria, private universities appeared to be the first to embrace virtual learning, particularly to avoid a total disruption of academic activities during the COVID‐19 pandemic and later became their major platform to facilitate learning (Egoigwe, 2020). Some of the e‐learning platforms explored during this period include Zoom, Microsoft Teams, Google Hangout (meet), Skype, Bamboo learning, Google classroom, Docebo, WIZIQ, Adobe Captivate, Elucidate and Blackboard Collaborate (Adeoye et al., 2020). Microsoft Teams users were reported to be 750 as of 10 March 2020 but by 24 March 2020 it has risen to 138,698, which is indeed an exponential growth. (OECD, 2020). This growth was also seen with the Zoom application which has necessitated an increase in video conferencing time in countries like Italy, Japan, United States and China (Molla, 2020).

Japan has recently used robotics to conduct a virtual graduation ceremony for their students, which is a clear indication of the unpredictable level of development in computer technology evident in the innovative development of advanced countries (Kacerauskas & Kusaityte, 2020). Despite all these innovations, there has been an urgent need to assess the impact of virtual learning sessions on learners as the world progresses in this system of learning. Holley (2002) argued that students who participate in virtual learning activities got better grades than students who studied through the conventional approach; thus, a virtual learning system is now being adopted by several higher institutions. This finding is confirmed by a more recent study carried out in Indonesia by Bali and Liu (2018). Similarly, Mogus et al. (2012) discovered that virtual learning activities had a positive impact on the final mark of the students under study. Adeoye et al. (2020) maintained that if universities start using a virtual learning system, it may lead to knowledge efficiency in students and lecturers due to the ease of access to enormous information within the globe. He also discovered that the problem of learning space will be solved and there would be room for learner‐to‐learner interaction and learner‐to‐teacher interactions. Pingle (2011) discovered that Indian undergraduates were more comfortable working with computers and other virtual learning programmes than the conventional face‐to‐face mode of learning. There is a dearth of literature in Nigeria that examined the impact of virtual learning from the learner's perspective especially in nursing education. Therefore, this study intends to assess the perceived impact on learners of virtual learning experiences during an online research conference organized by the Institute of Nursing Research, Nigeria during the COVID‐19 pandemic in May 2020 as a case study.

## 2 OBJECTIVES

The general objective of this study is to assess nurses and nursing students' perceptions of the impact of virtual learning.

### 2.1 Specific objectives

- To assess the perceived impact of a virtual research conference during the COVID‐19 lockdown.
- To assess participants' level of satisfaction with a virtual research conference during the COVID‐19 lockdown.
- To determine the relationship between the perceived impact of the programme and the various learning interactions.

### 2.2 Research hypothesis

There is no statistically significant correlation between learner–learner interaction, learner‐content interaction, learner–instructor interaction and the perceived impact of the programme.

## 3 METHODS

### 3.1 Study design

The study utilized a descriptive quantitative design using a structured online survey. Participants were recruited from a week‐long virtual nursing conference organized by the Institute of Nursing Research, Nigeria. The conference was held via Zoom and WhatsApp due to the COVID‐19 lockdown and the participants were recruited after each session. The conference aimed to improve nurses' knowledge about the research process and dissemination of findings. During the conference, different speakers who are experts in the nursing and research field facilitated training sessions and the participants were able to discuss and ask questions. The conference spanned over 7 days (Sunday, May 10, 2020, to Saturday, May 16, 2020), see Appendix 1 (conference order of event). As Zoom meetings were just getting popular during the early period of the lockdown, we held two testing sessions on Saturday, May 9, 2020, a day before the conference and on the first day of the conference to attend to all technical issues from the moderators and the participants. There was only an evening session on Sunday, May 10, 2020, other days had a morning and evening session, and an afternoon session was added to the sessions on Tuesday and Wednesday. Some of the theme sessions were titled ‘Selecting a Research Topic: The Dos and Don'ts; The Dynamics of Oral, Poster, and Paper Presentation; Theories in Nursing Research; Publish and Flourish‐ The A‐Z of Writing for Publication; Research Design and Data Analysis’.

### 3.2 Participants

In this study, 236 participants were recruited. Some of the participants were nursing students studying in various accredited institutions of learning and nursing staff practicing in various facilities in Nigeria and other countries.

### 3.3 Outcomes

Three outcomes were the focus of this study. They include satisfaction with the virtual conference, perceived impact of the conference and relationship between impact and learning interaction.

### 3.4 Instruments for data collection

Data were collected through an online survey using a 35‐item questionnaire adapted from the Student Satisfaction Survey (Strachota, 2003). This instrument assessed the satisfaction of students with a programme using different domains. The first was the learner‐content interaction. This is a learner's interaction with the learning content including PowerPoint presentation content, videos, website articles, quote posters, reports from rapporteurs, Zoom chats, WhatsApp texts and pictures. The second domain is the learner–instructor interaction. This refers to communication between the learner and instructor during a learning session. For this conference, this was ensured through the question‐and‐answer session, interaction through the Zoom chat and audio function, and WhatsApp messages between instructors and the participants. The third domain is the learner–learner interaction, which refers to communication between learners during a learning session. For this conference, this was ensured through WhatsApp and Zoom chats between conference participants. The last domain is the learner‐technology domain, which refers to the interaction of the learners with the technology used for learning. For this conference, the technologies participants interacted with included PowerPoint slides, Zoom and WhatsApp.

The instrument for data collection had seven sections, see Appendix 2 [questionnaire]. The first section (section A) elicited information based on socio‐demographics and this section had four questions. Section B contained 4 questions from the learner‐content interaction domain; Section C contained 4 questions that focused on the learner–instructor domain; Section D contained 3 learner–learner interaction questions; Section E contained 10 questions on learner‐technology interaction and Section F contained 4 questions which focused on the perceived impact of the programme. A 5‐point Likert scale was used to assess this. Strongly agree = 5, Agree = 4, Neutral = 3, Disagree = 2, Strongly disagree = 1. A total of 20 points was made for the questions, the scores were subsequently graded to range between 4 and 20. <9 was low, 10–15, moderate and >14, high. The lower scores represent lower satisfaction and vice versa. Section G contained 6 questions on the level of satisfaction of participants with the online programme. The instrument for data collection adopted from Strachota (2003) was designed from the typology of online interaction by Moore and Kearsley (1996) with a Cronbach alpha coefficient of 0.9, which indicates a satisfactory internal validity and reliability of the instrument.

### 3.5 Participant recruitment

During the first day of the conference, the 278 conference participants were informed about the research. Inclusion criteria included: (i) nursing students studying nursing at institutions accredited by the Nursing and Midwifery Council of Nigeria (NMCN), (ii) practising nurses licensed to practise by NMCN and (iii) registration and participation in the conference. Exclusion criteria included: (i) those who declined to participate in the research, (ii) those who did not register for the conference and (iii) those who did not fully participate in the conference.

An electronic information sheet containing details of the research was sent to the 240 participants that met the inclusion criteria. They were informed that participation in the study was voluntary, and they were free to decline to participate at any point. After they completed the consent form, they were directed to the survey link. Eventually, we got 236 responses, which translated to a response rate of 98.3%.

### 3.6 Ethical consideration

Permission to collect data was obtained from the ethical review board of Ladoke Akintola University of Technology Teaching Hospital, Osogbo. Participants were aware that participation in the study was voluntary and without any monetary reward. Information about their right to withdraw from the study was also provided. The principles of anonymity and confidentiality were also maintained. Informed consent was obtained from each participant, and no one was coerced into participating.

### 3.7 Data collection

Who registered for the virtual conference were invited to join the study. They received an invitation email that contained the link to the electronic version of the questionnaire and consent form. Those who consent to join the study filled the questionnaire and submitted the questionnaire through the same link that was provided to them. To ensure that all parts of the questionnaire were completed, each question carried the required sign such that the respondent could not submit the whole questionnaire until all questions are answered. Data was collected from 236 participants of the virtual research conference organized by a research institute based in Nigeria to assess the impact of the conference and the level of satisfaction of the participants during a pandemic. Respondents were allowed to choose either to participate in the research or not.

### 3.8 Method of data analysis

At the end of the conference, the retrieved questionnaires were sorted, cross‐checked and serially coded. Data entering, cleansing and analysis were done using IBM SPSS (version 23). Descriptive statistics including frequencies and percentages were used to summarize the data. To assess the association between different domains of interaction and the perceived impact of the programme, a multiple linear regression was conducted. In this study, the overall Likert scale response was converted into percentages to ensure uniformity in the scores.

## 4 RESULTS

Well over 500 nurses and nursing students registered for the virtual conference, 250 attendees of the conference agreed to participate while 236 duly filled questionnaires were submitted.

### 4.1 Participants' characteristics

Most of the participants are Nigerians (98.7%), between the age of 21 years and 30 years (60.6%). 38% had BSC and 15.7% has RN while others have or in the process of obtaining higher degrees. The table also shows that many of the participants were internet compliant given that software and applications are most common to people in this age bracket, and it is of no surprise that they dominate the conference. The average age of participants in this study is 23 years, 1.7% represent the minority of the participants 51–60 years. See Table 1 below.

**TABLE 1:** Socio‐demographic data of the participants.

Demographics	Frequency (*n* = 236)	Percentage (%)
Nationality		
Nigerian	233	98.7
Non‐Nigerian	3	1.27
Age (Average age 23.8 ± SD)		
≤20	58	24.6
21–30	143	60.6
31–40	25	10.6
41–50	6	2.5
51–60	4	1.7
Education		
BNSc	90	38.1
BNSc in view	57	24.2
RN	37	15.7
RM	2	0.8
RN in view	8	3.4
MSc	12	5.1
PGDE	1	0.4
PhD	1	0.4
Post Basic	4	1.7
Tertiary	24	10.2

### 4.2 Conference structure and the perceived impact of virtual learning

The impact of the virtual learning was assessed based on the learner–learner, learner‐content and learner–instructor scale of the questionnaire. In the learner‐content scale, the mean score was 17.06 of a possible 20 points, which when further stratified into levels, resulted in most of the response (91.1%) favouring a high level of learner‐content interaction. This implies that most of the participants scored this domain with higher than 14. The second domain which is the learner–instructor interaction, the mean score was 14.87 of a possible 20 points, which when further stratified into levels, resulted in over an average (55.1%) of the participants who scored high in this domain, 43.6% of the participants thought the interaction was moderate, only a few of them (1.3%) claimed that it was low. Furthermore, the third domain was the learner–learner interaction, the mean score was 10.44 of a possible 15 points, which when further stratified into levels, resulted in just above half (51.7%) of the participants scoring high while a lower percentage (44.1%) scored this domain as moderate and few of them scored low. The level of satisfaction of each participant was assessed and evaluated, a lot of the participants (75.4%) were highly satisfied with the conference, 24.2% were moderately satisfied and 0.4% considered their satisfaction to be low. Lastly, on the perceived impact of the programme, 89.9% perceived the programme to be highly impactful, while 7.6% perceived the programme to be moderately impactful. 2.5% perceived the programme had a low impact. See Table 2 below.

**TABLE 2:** Domains of impact and levels of satisfaction with the virtual conference.

	Frequency (*n* = 236)	Percentage (%)
Learner‐content interaction		
Mean score—17.06 ± 2.54		
High (>14)	215	91.1
Moderate (10–14)	16	6.8
Low (<9)	5	2.1
Learner–instructor interaction		
Mean score—14.87 ± 2.17		
High	130	55.1
Moderate	103	43.6
Low	3	1.3
Learner–learner interaction		
Mean score—10.44 ± 0.2.51		
High	122	51.7
Moderate	104	44.1
Low	10	4.2
Level of satisfaction		
High	178	75.4
Moderate	57	24.2
Low	1	0.4
Perceived impact of the programme		
High	212	89.8
Moderate	18	7.6
Low	6	2.5

Association between the conference structure and the perceived impact of virtual learning.

Multiple linear regression analysis was conducted to determine the relationship between the conference structure and the perceived impact of the conference. The result revealed a statistically significant positive relationship between the Learner–learner Interaction (*p =* 0.020), Learner‐Content Interaction (*p =* 0.000), Learner–instructor Interaction (*p =* 0.000) and perceived impact of the programme (Table 3).

**TABLE 3:** Multiple linear regression showing an association between different domains of interaction and perceived impact of the programme.

	Unstandardized B	Coefficients SE	Standardized coefficients Beta	*t*	Sig.
Constant	4.368	1.091		4.005	0.000
Learner–learner interaction	0.130	0.055	1.091	2.339	0.020
Learner‐content interaction	0.408	0.057	0.411	7.165	0.000
Learner–instructor interaction	0.284	0.068	0.245	4.194	0.000

Table 4 shows that the multiple linear regression model predicts 37.2% of the overall variance in the perceived impact of the programme and this is statistically significant using the *f*‐test (*p* = 0.02). All the independent variables (learner‐content interaction, learner–instructor interaction and learner–learner interaction) are statistically significant predictors of the dependent variable (perceived impact of the programme) at *α* = 0.05 level of significance.

**TABLE 4:** Model summary of the predicting factors influencing the overall perceived impact of the programme.

Model	*R*	*R* ^2^	Adjusted *R* ^2^	SE of the estimate	*R* ^2^change	*F* change	df1	df2	Sig. *F* change	Durbin‐watson
1	0.541^a^	0.293	0.290	2.12557	0.293	96.925	1	234	0.000	
2	0.598^b^	0.357	0.352	2.03120	0.064	23.247	1	233	0.000	
3	0.610^c^	0.372	0.372	2.01199	0.015	5.471	1	232	0.020	1.866

^a^ Predictors: Constant, Learner‐content interaction.

^b^ Predictors: Constant, Learner‐content interaction, Learner–instructor interaction.

^c^ Predictors: Constant, Learner‐content interaction, Learner–instructor interaction, Learner–learner interaction.

^d^ Dependent variable: Perceived impact of the programme.

## 5 DISCUSSION

This research investigated the perceived impact of virtual learning using the online research conference organized by the Institute of Nursing Research, Nigeria during the COVID‐19 pandemic in May 2020 as a case study. Given the novelty of this teaching mode especially to the Nigerian nurses and nursing student population, it was necessary to assess the satisfaction and impact of the conference on learners. We believe that the evidence generated from this study would further inform future designs of virtual learning programmes for the Nigerian nursing population.

The pandemic created a gap that started since COVID‐19 was declared a public health emergency concern on 30 January 2020. Technology served as an aide to help bridge the gap. Classes, conferences and seminars moved to digital space to limit the effect of disruption of regular events and crowd restrictions. This study focuses on the perceived impact of virtual learning during COVID‐19 lockdown. Nearly all the respondents were middle‐aged with at least a diploma and/or a bachelor's in nursing. The design of the data collection instrument was based on five domains: learner‐content interaction, learner–instructor interaction, learner–learner interaction, level of satisfaction and perceived impact.

Learner‐content interaction refers to concepts and facts that are expected to be learnt and how learners relate with them (UNESCO‐IBE, 2013), including having classes in line with the learning objectives. This can be in the form of PowerPoint or audio or video aides. The study indicated that nearly all the respondents believed the conference had a high learner‐content interaction, which totally disagrees with a previous study that distance learning using online medium does not meet the learning outcomes of its learner (Xiao, 2017) and agrees with (Turley & Graham, 2019) that using online medium gives high learner‐content interaction.

Learner–instructor interaction is communication between participants and teacher in a course; this helps build participants' skills and self‐esteem. More than half of the participants believed that the online conference provided a high level of learner–instructor interaction. This implies that online learning platforms could stimulate nurses and nursing students' interest and confidence in relating with nurse educators to ensure a good learning culture and outcomes. In a previous study by Zimmerman (2012), participants with high learner–instructor were found to have better performance and satisfaction. Another survey by Hunter and Ross (2019) showed that increased online interaction between instructors and students positively affected students' perception of the course quality.

Learner–learner interaction refers to interaction with other learners enrolled in the same course via communication methods such as discussion boards, video messages and collaborative class projects. About half the participants indicated that the online conference had a high level of learner–learner interaction as they could relate with other attendees of the meeting via discussions, breakout sessions and WhatsApp chat room. Hunter and Ross (2019) demonstrated that students' perception of the quality of a course statistically significantly depends on the learner–learner interaction. Jason and Jason (2006) suggested that the quality of online education should improve with a renewed focus on incorporating learner–learner interaction. This reveals the importance of learner–learner interaction in designing and implementing any virtual or online course.

Three‐quarter of the respondents indicated a high level of satisfaction towards the conference. Bramer (2020) discovered a high level of satisfaction among student nurses as online clinical skill courses and materials helped them achieve better practice in the clinical area.

From testing the hypothesis: there is no statistically significant association between learner–learner interaction, learner‐content interaction, learner–instructor interaction and the perceived impact of the programme. All three domains: learner‐content interaction, learner–instructor interaction and learner–learner interaction, were found to have a statistically significant association with the perceived impact of the programme. This implies that each domain goes a long way in determining the effects of online courses designed for nurses and nursing students. The multiple regression model predicted 37.2% overall variance in the perceived impact of the programme, thus indicating that the three domains tested are statistically significant predictors of the perceived impact of the programme. This finding is consistent with several authors who discovered that nursing and midwifery students indicated more satisfaction with courses taken only when the platform availed them good interaction with the course contents, with their lecturers and with other nursing students on the same course (Bramer, 2020; Daly et al., 2019; Egoigwe, 2020).

## 6 CONCLUSION

The findings of this study showed that the perceived impact of the virtual conference is associated with the interactions of participants with the content, instructors or facilitators and other participants. Aside the impact, the ease of access to the conference was evident in the number of attendees implying that the use of virtual platforms can promote attendance of nurses and nursing student to various trainings and conferences. However, future studies need to consider identifying cost, accessibility and flexibility issues that may be related to utilizing virtual platforms for trainings and conferences. Additionally, the differences in the view of nurses and student nurses with respect to the impact of virtual platforms on learning can be researched by future studies. While the return to normalcy in the foreseeable future is anticipated, it is recommended that trainings and conferences for nurses should embrace virtual learning platforms alongside the traditional face‐to‐face training or conference modes. Future studies may consider comparing the effects of different virtual platforms on the learner‐content, learner–instructor and learner–learner interaction and the satisfaction of learners with each platform.

## AUTHOR CONTRIBUTION

Each author contributed equally to the design, data collection, data analysis and writing of this manuscript.

## ACKNOWLEDGEMENTS

We sincerely appreciate the leadership of the Institute of Nursing Research, Nigeria for initiating this study, supervising and supporting the scholars throughout the project.

## FUNDING INFORMATION

This research did not receive any specific grant from funding agencies in the public, commercial or not‐for‐profit sectors.

## CONFLICT OF INTEREST STATEMENT

The authors declare that they have no known competing financial interests or personal relationships that could have influenced the work reported in this paper.

## Data Availability

The data that supports the findings of this study are available in the supplementary material of this article.

## APPENDIX 1

### ORDER OF EVENTS

Day 1

Saturday, 9th May, 2020

**Table:**

**04:00 PM**	**Zoom Testing Session**

Day 2

Sunday, 10th May, 2020

Moderator: Adefehinti Pelumi—INR, Nigeria

**Table:**

**02:00 PM**	**Welcome address and Pre‐Test (WhatsApp Session)**
**04:00 PM**	**Zoom Testing Session II**
**05:00 PM**	**Overview of Research Process—** *Dr Ruth Ifediora*, *President*, *Comfort Foundation*, *US* **(Zoom Session)**

Day 3

Monday, 11th MAY, 2020

Moderator: Aladegbami Damilola (NUNSA BOWEN University)

**Table:**

**10:00 AM**	**Research without Tears: Building the Competencies of the 21st Century Nurse in Research and Scholarly Endeavours**—*Prof Prisca Adejumo*, *Head*, *Department of Nursing*, *U*.*I* **(Zoom Session)**
**07:00 PM**	**Selecting a Research Topic: The Dos and Don'ts**—*Faleti Damilola—INR*, *Nigeria* **(WhatsApp Session)**

Day 4

Tuesday, 12th May, 2020

Moderator: Shado Olamide (INR Nigeria)

**Table:**

**10:00 AM**	**Nurses: A Voice to Lead in Clinical Research—** *Prof*. *Bayo Ajibade*, *Dean*, *Faculty of Nursing*, *LAUTECH* **(Zoom Session)**
**02:00 PM**	**The Dynamics of Oral, Poster and Paper Presentation—** *Dr*. *Ngozi Emilia Iwu*, *President*, *National Association of Nigerian Nurses in North America* **(Zoom Session)**
**07:00 PM**	**Nurses and SDGs: Relevance of Nursing Research Towards Achieving SDGs, UHC and Africa 2063 Agenda—** *President*, *Africa Novice Nurses and Students Initiative*, *Egypt* **(Zoom Session)**

Day 5

Wednesday, 13th May, 2020

Moderator: Ogundipe Mabel—INR Nigeria

**Table:**

**10:00 AM**	**Theories in Nursing Design—** *Tola Yetunde*, *The Nethersole School of Nursing*, *CUHKj*, *Hong Kong* **(Zoom Session)**
**02:00 PM**	**Role and Relevance of Nursing Research in Achieving Nursing Now Global Campaign—** *Oluyemisi Adegbile*, *NNCA UK* **(Zoom Session)**
**07:00 PM**	**Literature Review‐ Techniques, Skills and Pitfalls—** *Faleti Daniel*, *INR*, *Nigeria*. **(WhatsApp Session)**

Day 6

Thursday, 14th May, 2020

Moderator: Joshua Emmediong (Post Basic Ophthalmic Nursing School, UBTH)

**Table:**

**10:00 AM**	**Publish and Flourish‐ The A‐Z of Writing for Publication—** *Akingbade Damilare*, *INR*, *Nigeria* **(Zoom Session)**
**07:00 PM**	**The 21st Century Nurse: Building Your Personal Nursing Brand—** *Adewa Taiwo*, *INR Nigeria* **(WhatsApp Session)**

Day 7

Friday, 15th May, 2020

Moderator: Kolawole Oluwatobi (INR Nigeria)

**Table:**

**07:00 PM**	**Data Analysis I**—*Adesuyi Emmanuel*, *INRN*, UK **(Zoom Session)**
**10:00 AM**	**Research Design—** *Dr*. *Bunmi Ogungbe*, *Johns Hopkins University*, *U*.S **(Zoom Session)**

Day 8

Saturday, 16th May, 2020

Moderator: Dairo Emmanuel (INR, Nigeria)

**Table:**

**10:00 AM**	**Data Analysis II—** *Adesuyi Emmanuel*, *INRN*, UK **(Zoom Session)**
**02:00 PM**	**Post Test (WhatsApp Session)**
**07:00 PM**	**Establishment of INR Research Clubs in Nigerian Nursing Citadels of Learning—** *Fawole Israel—NUNSA National President* **(Zoom Session)**
	**Closing Remarks and Award of Certificates** **(WhatsApp Session)**
